# “To do, or not to do?”: determinants of stakeholders’ acceptance on dengue vaccine using PLS-SEM analysis in Malaysia

**DOI:** 10.1186/s12889-022-13967-3

**Published:** 2022-08-19

**Authors:** Ahmad Firdhaus Arham, Latifah Amin, Muhammad Adzran Che Mustapa, Zurina Mahadi, Mashitoh Yaacob, Ahmad Fadhly Arham, Nor Sabrena Norizan

**Affiliations:** 1 Pusat Pengajian Citra Universiti, Universiti Kebangsaan Malaysia (CITRA UKM), UKM Bangi, Selangor Malaysia; 2 The Institute of Islam Hadhari (HADHARI), Universiti Kebangsaan Malaysia, UKM Bangi, Selangor Malaysia; 3 Faculty of Business and Management, Universiti Teknologi Mara (UiTM), Melaka, Malaysia

**Keywords:** Dengue vaccine, Attitude, Intention, Scientists, Public, PLS-SEM, Malaysia

## Abstract

**Background:**

Dengue vaccine is a promising alternative for protecting communities from dengue. Nevertheless, public acceptance of the dengue vaccine must be considered before the authorities decide to carry out intensified research and recommend the vaccine adoption. This study aimed to assess the stakeholders' acceptability of the dengue vaccine and determine the factors that influence their intentions to adopt it.

**Methods:**

Survey data collected from 399 respondents who represented two primary stakeholder groups: scientist (*n* = 202) and public (*n* = 197), were analysed using the partial least squares-structural equation modelling (PLS-SEM) technique.

**Results:**

The findings revealed that the stakeholders claimed to have a highly positive attitude and intention to adopt the vaccine, perceived the vaccine as having high benefits, and displayed a high degree of religiosity and trust in the key players. The results also demonstrated that attitude and perceived benefits significantly influenced the intention to adopt the dengue vaccine. Furthermore, the perceived benefit was the most significant predictor of attitude to the dengue vaccine, followed by religiosity, attitudes to technology, and trust in key players.

**Conclusion:**

The findings showed that the stakeholders in Malaysia were optimistic about the dengue vaccine with a positive attitude and perceived benefits as significant predictors of intention to adopt the vaccine. Hence, ongoing research can be intensified with the end target of recommending the vaccine for public adoption in hotspot areas. This finding contributes to the consumer behaviour literature while also providing helpful information to the government, policymakers, and public health officials about effective strategies for driving dengue vaccine acceptance in Malaysia and other countries with a history of severe dengue transmission.

**Supplementary Information:**

The online version contains supplementary material available at 10.1186/s12889-022-13967-3.

## Introduction

Dengue is no longer a rare disease because dengue cases have been on the rise globally including in Malaysia. The disease poses a threat to health and the economy in tropical and subtropical countries [1]. The main vectors responsible for the dengue disease are *Aedes aegypti* and *Aedes albopictus.* Besides, these mosquitoes are also responsible for chikungunya and Zika viruses [2]. Several serotypes of dengue diseases are DENV 1, DENV 2, DENV 3, and DENV 4. There are many current approaches to combat dengue, such as fogging, indoor and outdoor residual spraying, the release of the male *Wolbachia*-infected *Aedes* mosquitoes, the development of genetically modified *Aedes* mosquitoes, and others. These approaches were the current technology in use, and some are currently in research to reduce all dengue virus serotypes in Malaysia. However, the dengue vaccine development is a promising approach to protect the community from dengue.

After decades of research by Sanofi Pasteur, the first dengue vaccine, Dengvaxia® (CYD-TDV), was first licensed in Mexico in December 2015 for individuals between 9–45 years old, living in endemic areas. The vaccine is now available in 20 countries [3] and has been used in large-scale vaccination programmes in the Philippines, engaging over 800,000 school children [4]. Dengvaxia® has the potential to reduce the dengue burden in endemic populations due to its cost-effectiveness, efficacy, and user-friendly feature [5]. According to Pasteur's research, the vaccine is more effective and is encouraged to be injected into people who have been infected with the disease [6].

Flasche et al. [7] showed that dengue vaccine implementation would reduce dengue symptoms and hospitalisation rate by 13% to 25% in the first 30 years after vaccination. Although Shim [8] indicated that age-targeted Dengvaxia® vaccination is cost-effective in Brazil, the results indicated that routine vaccination of 70% of nine-year-olds reduces the dengue infection by 79% and if the targeted age group widens, the cost-effectiveness is reduced. Espana et al. [9] also discovered that the vaccine could reduce severe dengue by preventing 5.5% of hospitalisations. Besides, their findings also revealed that this intervention could be cost-effective in Puerto Rico at the cost of 382 USD. Moreover, herd immunity from Dengvaxia® promises a sense of security and safety from dengue disease [9]. Dengvaxia® has 66% efficacy, which could benefit public health and economics because the protection level is considerable [10]. However, there is still a need for more research on a dengue vaccine that will be effective for all age groups.

Despite the vaccine’s potential, Malaysia has conditionally approved the vaccine for testing despite the vaccine's potential, but it has not been fully implemented. So, it is important to study the public acceptance of this new approach before its adoption. In Malaysia, Yeo and Shafie [1] researched the public’s acceptance of the dengue vaccine to determine their willingness to pay for the vaccine, the respondents from Pulau Pinang positively reacted to the dengue vaccine and indicated their willingness to pay for the vaccine for the sake of their health. In another research, Arifah et al. [11] showed that health workers in Klang Valley were willing to pay between RM1 to RM500 (0–120 USD) for the dengue vaccine. Thus, their willingness to pay for the vaccine shows their acceptance of the vaccine.

Therefore, this study supports the studies mentioned above and a follow-up from the study of Arham et al. [12, 13], who examined stakeholders' acceptance of Outdoor Residual Spraying and W*olbachia*-infected *Aedes* mosquitoes' techniques, which indicated that they positively support the approaches. Hence, a study focusing on the stakeholders' acceptance of the dengue vaccine and its predictors is also needed. Therefore, the main objective of this study is to determine the Malaysian stakeholders' acceptance of the dengue vaccine and determine its predicting factors. The finding will contribute to the existing literature on consumer behaviour toward adopting dengue vaccines. While also provides valuable information to the government, policymakers, and public health officials about effective strategies for driving dengue vaccine acceptance in Malaysia and other countries with a history of severe dengue transmission.

### Theory and research hypotheses

The model theory of this study was developed and adapted based on the study by Amin and Hashim [14] which was developed from Fishbein’s attitude model. Amin and Hashim’s model became the main reference in determining the predictor factors influencing attitudes towards genetically modified mosquitoes as one of the dengue control techniques [14]. Therefore, four components proposed in the research model of this study include general factors, specific factors, attitude, and intention. General factors are predictive factors consisting trust in key players, attitudes to technology and religiosity. Previous studies tested all these factors as general factors in determining stakeholders’ acceptance of dengue controlling techniques [12–17]. These general factors have been observed to play a crucial role in directly and indirectly determining a person’s attitude and intention. Nevertheless, these general factors have been initially pioneered through past studies for trust in key players [18–25], attitudes to technology [21, 26–28], and religiosity [26, 27].

Specific factors, namely perceived benefit and perceived risk are predictive factors. Both of these factors have made clear direct contributions to determine attitude and intention towards dengue controlling techniques in past studies [12–17]. These two factors play significant roles by being an essential basis directly related to the formation of attitude and intention in past studies. These factors are commonly known to have an inverse relationship in determining attitude and intention [28–34]. Attitude and intention are components that determine the views, acceptance, or approval to express support for something. Attitude represents beliefs that describe actions to behave based on positive or negative intention [35–37].

**Fig. 1:**
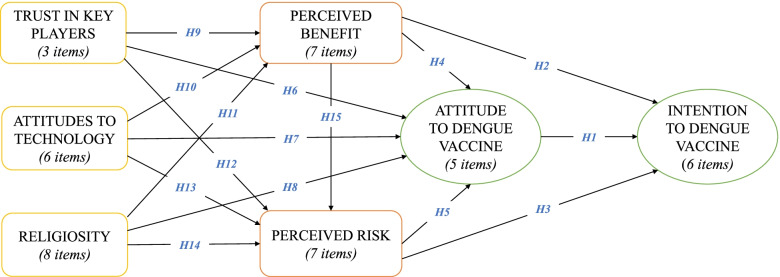
Research conceptual framework

The hypotheses were developed based on the Pearson correlation method [38]. Therefore, 15 hypotheses were developed according to the study’s framework to determine the relationship of predictor factors with the attitude and intention of stakeholders’ acceptance of the dengue vaccine (Refer to Fig. 1).

*H1: Attitudes has a significant influence on intention among stakeholder to adopt the dengue vaccine*

*H2: Perceived benefit has a significant influence on intention among stakeholders to adopt the dengue vaccine*

*H3: Perceived risk has a significant influence on intention among stakeholders to adopt the dengue vaccine*

*H4: Perceived benefit has a significant influence on attitude among stakeholders to adopt the dengue vaccine*

*H5: Perceived risk has a significant influence on attitude among stakeholders to adopt the dengue vaccine*

*H6: Trust in key players has a significant influence on attitude among stakeholders to adopt the dengue vaccine*

*H7: Attitude to technology has a significant influence on attitude among stakeholders to adopt the dengue vaccine*

*H8: Religiosity has a significant influence on attitude among stakeholders to adopt the dengue vaccine*

*H9: Trust in key players has a significant influence on perceived benefit among stakeholders to adopt the dengue vaccine*

*H10: Attitude to technology has a significant influence on perceived benefit among stakeholders to adopt the dengue vaccine*

*H11: Religiosity has a significant influence on perceived benefit among stakeholders to adopt the dengue vaccine*

*H12: Trust in key players has a significant influence on perceived risk among stakeholders to adopt the dengue vaccine*

*H13: Attitude to technology has a significant influence on perceived risk among stakeholders to adopt the dengue vaccine*

*H14: Religiosity has a significant influence on perceived risk among stakeholders to adopt the dengue vaccine*

*H15: Perceived benefit has a significant influence on perceived risk among stakeholders to adopt the dengue vaccine*

### Methodology

#### Study design, location, and duration

A close-ended multidimensional survey instrument was designed to identify factors influencing stakeholders’ acceptance of the dengue vaccine in Klang Valley, Malaysia. The instruments used in this study consist of seven variables: 1) trust in key players, 2) attitudes to technology, 3) religiosity, 4) perceived benefit, 5) perceived risk, 6) attitude and 7) intention to dengue vaccine. The items used were adapted and modified from previously published work by Amin and Hashim [14] and previous studies [18–27]. Klang Valley was chosen as the location of the study because this area is the hotspot of dengue cases in Malaysia (http://idengue.arsm.gov.my) and the center of socio-economic development.

The questionnaire was developed in Malay and translated into English to allow respondents to choose to respond in a language that they were more comfortable. Certified translators validated the two-way translation. Respondents were asked to evaluate their opinion on a 7-point Likert scale ranging from 1 (strongly disagree) to 7 (strongly agree) for each item in this instrument. According to Churchill and Dawn [39], Likert-scale questionnaires need to have many options so that the respondents can give the closest answer and represent themselves. Likewise, Wu and Leung [40] also reported that an increased number of Likert-type scale points will result in a closer approach to the underlying distribution, hence normality and interval scales.

Experts in environmental health, social science, and governance examined the content and face validity of the questionnaires. Prior to the actual study, 126 questionnaires were distributed for a pilot study to test the strength of the items used and determine the research instruments' validity and reliability. After the pilot study, an exploratory principal component factor analysis followed by varimax rotation was carried out to identify items best expressive of attitudinal dimensions. The items which cross-loaded on more than two factors and were difficult to interpret, with factor loadings lower than 0.50 or inconsistency, were deleted. The enumerators continue to distribute the questionnaire from September 2016 to September 2017.

### Ethics statement

Before the study's procedures, participants consented verbally and voluntarily, and all was done following the Declaration of Helsinki and the Malaysian Ministry of Health's Medical Review & Ethics Committee (MREC). Therefore, ethical approval was not required for this study since under the Guidelines for Ethical Review of Clinical Research or Research involving human subjects, Medical Review and Ethics Committee [2006] (www.nccr.gov.my/index.cfm?menuid=26&parentid=17), research involving questionnaires with no collection of identifiable private information is exempted from review by the Medical Review and Ethics Committee.

### Sample size, participation, and data collection

Faul et al. [41] suggested conducting statistical analysis for social and behavioural sciences using the G*Power 3.1.9.2 software. This software used a linear multiple regression test to determine the sample size using statistical power of 0.80 [42], medium-size effect (*f* = 0.15), and significance level (*p* = 0.05) with 15 paths of exogenous latent variables representing 15 hypotheses predicted to have an impact in the research conceptual framework model. The analysis indicated that this study only required 139 respondents. Therefore, this study also considers the total population located in the Klang Valley and the number of dengue cases in 2015, which recorded 23,355 dengue cases reported by OR Technologies, Malaysia (https://public.tableau.com/app/profile/ortechnologies/viz/KadarKesKematianAkibatDenggi2010-2015hackathon2/KadarKesKematianAkibatDenggi2010-2015).

Using stratified random sampling, this survey was undertaken face-to-face among 415 Malaysian adults (aged 18 years and above). However, only 399 respondents were analysed after validity and reliability screening due to complete responses and no biased. Krejcie and Morgan [43] proposed a total sample size of 384 respondents for over 1 million population. Hence, the total sample of respondents for scientists and the public in this study is considered sufficient. The respondents were initially divided into two groups: scientists (*n* = 202) and the general public (*n* = 197). The two groups were merged for analysis as they share a common interest in adopting the dengue vaccine. Academicians, postgraduate students, research officers working in environmental science, biological sciences, health, and genetic sciences research, and those participating in dengue control and prevention are categorised as scientists. The public consists of people living in outbreak regions in the Klang Valley, classified as areas with high *Aedes* mosquito numbers. The participation of the respondents was voluntary. Nevertheless, informed consent was obtained verbally, and the respondents’ details were kept confidential.

### Data analysis

Partial Least Square Structural Equation Modelling (PLS-SEM) using the Smart Partial Least Square (Smart-PLS) software version 3.3.9 was employed to assess the hypothesised relationships [44]. This approach is particularly beneficial in justifying the interaction between multiple factors to explain complicated behaviour [45]. Firstly, the measurement model was investigated to determine the validity and reliability. Subsequently, the structural model was tested to test the hypotheses, including the model fit test [46, 47]. In addition, a bootstrapping approach with 5000 resamples was utilised to determine the relevance of the path coefficient and loading. A normality test for statistical analysis was also performed to confirm that the data did not cut off the normality criterion [45, 46, 48–50].

## Findings and discussions

﻿The summary socio-demographic characteristics of the sample are presented in Table 1. The respondents were 197 scientists and 202 public, where 51.1% were female, and 48.9% were male. More than 70% of them were less than 40 years old. Approximately 42.4% of respondents were Malays, which reflected the actual population ratio in the Klang Valley, where most of them are Malays [51]. Table 2 shows the overall mean scores for religiosity (﻿with a mean score of 6.07), intention to dengue vaccine (with a mean score of 5.71), trust in key players (with a mean score of 5.51), attitude to dengue vaccine (with a mean score of 5.71), and perceived benefit (with a mean score of 5.38) were rated high. The stakeholders responded that they were entirely dedicated to their religion, trusted the key players, viewed the dengue vaccine as incredibly beneficial, and had a positive attitude and intention to accept it. Nevertheless, the stakeholders were rated moderate for attitudes to technology (with a mean score of 4.74, above the mid-point of 4.0) and perceived risk (with a mean score of 3.58, below the mid-point of 4.0). The findings imply that the stakeholders were more attracted to technology and believed that the dengue vaccination had limited risk.

**Table 1:** Profiles of respondents (*n* = 399)

Demographic Variables		Frequency	Percentage %
Type of Stakeholders	Scientists Public	197 202	49.4 50.6
Gender	Male Female	195 204	48.9 51.1
Age (years old)	18–28 29–39 Above 40	185 132 78	46.4 33.1 19.5
Race	Malay Chinese Indian Others	169 108 91 31	42.4 27.1 22.8 7.8

**Table 2:** Mean score and interpretation

*Factor*	*Mean* ± *Standard Deviation*	*Interpretation*
Intention to Dengue Vaccine	5.71 ± 1.02	High
Attitude to Dengue Vaccine	5.42 ± 1.00	High
Perceived Benefit	5.38 ± 1.08	High
Perceived Risk	3.58 ± 1.29	Moderate
Trust in Key Players	5.51 ± 0.94	High
Attitudes to Technology	4.74 ± 1.38	Moderate
Religiosity	6.07 ± 1.09	High

1.00–3.00, low; 3.01–5.00, moderate; 5.01–7.00, high

### Measurement model analysis

The analysis of the convergent reliability and validity of the variables is shown in Table 3. Convergent validity can be determined if the factor loadings are larger than 0.7 [52, 53], the composite reliability (CR) is more than 0.70 [54], and the average variance extracted (AVE) is larger than 0.50 [55, 56]. The findings indicated that the factor loadings of the items were higher than 0.7, except for several items (PBV1 = 0.693; PBV5 = 0.692; ATT1 = 0.698). Nonetheless, according to Byrne [55], if the total AVE exceeded 0.50, the factor loadings below 0.70 were retained. Therefore, all the variables had AVE values exceeding 0.50, and the values of CR were greater than 0.70, which is considered acceptable.

**Table 3:** Internal consistency and convergent validity

*Factor*	*Item*	*Loading*	*CR*	*AVE*	*Validity*
Intention to Dengue Vaccine	INT1 INT2 INT3 INT4 INT5 INT6	0.838 0.886 0.809 0.788 0.837 0.811	0.929	U0.687	YES
Attitude to Dengue Vaccine	ADV1 ADV2 ADV3 ADV4 ADV5	0.698 0.728 0.704 0.806 0.772	0.860	0.552	YES
Perceived Benefit	PBV1 PBV2 PBV3 PBV4 PBV5 PBV6 PBV7	0.693 0.773 0.775 0.787 0.836 0.692 0.714	0.902	0.569	YES
Perceived Risk	PRV1 PRV2 PRV3 PRV4 PRV5 PRV6 PRV7	0.762 0.773 0.802 0.789 0.799 0.775 0.793	0.918	0.616	YES
Trust in Key Players	TKP1 TKP2 TKP3	0.857 0.839 0.824	0.878	0.706	YES
Attitudes to Technology	ATT1 ATT2 ATT3 ATT4 ATT5 ATT6	0.782 0.867 0.898 0.900 0.895 0.804	0.944	0.738	YES
Religiosity	REG1 REG2 REG3 REG4 REG5 REG6 REG7 REG8	0.882 0.834 0.815 0.803 0.830 0.911 0.865 0.891	0.956	0.730	YES

AVE value must greater than 0.5; CR value must greater than 0.7

The discriminant validity analysis also found that the variables have met the requirements (Refer to Table 4). In the Fornell-Larcker criterion assessment, each variable has a more excellent square root value of AVE than the other variables [57]. The value of the Heterotrait-monotrait (HTMT) correlation for each of the variables was acceptable because the values were less than 0.85 [58, 59].

**Table 4:** Fornell-Larcker and HTMT Criterion

***Fornell-Larcker Criterion***							
	INT	ADV	PBV	PRV	TKP	ATT	REG
INT	0.829						
ADV	0.709	0.743					
PBV	0.601	0.558	0.755				
PRV	-0.077	-0.111	-0.108	0.785			
TKP	0.425	0.313	0.371	-0.311	0.840		
ATT	0.214	0.221	0.259	-0.346	0.183	0.859	
REG	0.321	0.310	0.190	-0.042	0.158	-0.031	0.855
***HTMT Criterion***							
	INT	ADV	PBV	PRV	TKP	ATT	REG
INT							
ADV	0.816						
PBV	0.670	0.658					
PRV	0.096	0.148	0.167				
TKP	0.503	0.391	0.445	0.353			
ATT	0.230	0.253	0.285	0.376	0.212		
REG	0.346	0.351	0.210	0.094	0.199	0.067	

The square root of the AVE value in the results was more than the total variance shared by the other variable factors. HTMT_0.90_ values do not exceed 1, indicating that the indicator for that factor is lower than the discriminant validity aspect

The measurement model analysis was also measured by standardised root mean square residual (SRMR) and normed fit index (NFI) as suggested by Lohmoller [60]. In accordance with the SRMR, when the values are below 0.8, it is considered as good model fit measure [61] while the NFI values higher than 0.9 are considered acceptable [46]. In this study, the SRMR value was 0.074, and the NFI value was 0.71, which was slightly lower than 0.9 (Refer to Table 5). However, the value is still within an acceptable range which is above 0.5 and closer to 1, a value considered an acceptable fit [62]. In addition, scholars also suggested to report the value of the root mean square error correlation (RMS_theta_) as the approximate model fit criteria [46, 60]. According to Henseler et al. (2014), the RMS_theta_ can distinguish between well-specified and ill-specified models [63]. The RMS_theta_ value was 0.11, lower than the threshold value of 0.12, indicating a well-fitting model [47]. The variance inflation factor (VIF) values for all the variables were lower than 5.0, suggesting no collinearity concerns the inner model [64].

**Table 5:** Good fit (SRMR and NFI value) and collinearity assessment

*Good Fit Assessment*				
SRMR (0.074); NFI (0.710)				
***Collinearity Assessment***				
	INT	ADV	PBV	PRV
ADV	1.457			
PBV	1.456	1.253		1.244
PRV	1.016	1.233		
TKP		1.280	1.064	1.184
ATT		1.215	1.038	1.092
REG		1.057	1.030	1.057

SRMR value below than 0.08; NFI value closer to 0.9; R^2^, VIF value must below 5.00

### Structural model analysis

The structural model analysis started with the coefficient of determination (*R*^*2*^) testing. The *R*^*2*^ value for the intention is 0.564, which shows that exogenous variables in the model could explain 56.4% of the variance in intention to dengue vaccine. The *R*^*2*^ value of the attitude is 0.371, suggesting that the exogenous variables explain 37.1% of the factor. Furthermore, the exogenous variables explained 19.6% of the variance in perceived benefit and 18.9% of the variance in perceived risk.

The analysis continued with the blindfolding procedure to measure the predictive accuracy of the model predictions (*Q*^*2*^), where the value must be beyond zero [65]. The *Q*^*2*^ values for the perceived benefit is 0.111, perceived risk is 0.109, attitude is 0.198, and intention to dengue vaccine is 0.383, which confirmed that the predictive relevance of the model was adequate for the exogenous variables. According to Cohen [66], attitude (*f*^2^ = 0.465) has a large effect size on intention to dengue vaccine compared with perceived benefit (*f*^2^ = 0.141). Perceived benefit has a medium effect size on attitude (*f*^2^ = 0.184), while the effect size of religiosity (*f*^2^ = 0.067), attitudes to technology (*f*^2^ = 0.012), and trust in key players (*f*^2^ = 0.011) was small. The findings also showed that trust in key players (*f*^2^ = 0.112), attitudes to technology (*f*^2^ = 0.051), and religiosity (*f*^*2*^ = 0.026) have a small effect size on perceived benefit. Lastly, attitude to technology (*f*^2^ = 0.113) and trust in key players (*f*^2^ = 0.082) have a small effect on perceived risk. Table 6 illustrates the results of *R*^*2*^, *Q*^*2*^, and *f*^2^ values.

**Table 6:** Determination of coefficient (R^2^), predictive relevance (Q^2^) and effect size (f ^2^)

	*Determination Coefficient*	*Predictive Relevance*	*Effect Size*					
	*R*^*2*^	*Q*^*2*^	ADV	PBV	PRV	TKP	ATT	REG
INT	0.564	0.383	0.465 (Large)	0.141 (Small)				
ADV	0.371	0.198		0.184 (Medium)		0.011 (Small)	0.012 (Small)	0.067 (Small)
PBV	0.196	0.111				0.112 (Small)	0.051 (Small)	0.026 (Small)
PRV	0.189	0.109				0.082 (Small)	0.113 (Small)	

R^2^, range from 0 to 1; f^2^, large ≥ 0.35, medium ≥ 0.15, small ≥ 0.02; Q^2^, greater than 0

### Direct relationships analysis

**Fig. 2:**
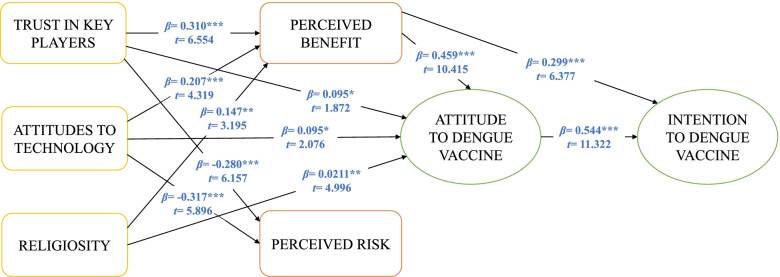
Model for stakeholders’ acceptance to adopt the dengue vaccine in Malaysia

The relationship between exogenous and endogenous variables was evaluated by examining the path coefficients’ size in the structural model. Attitude (*β* = 0.544, *t* = 11.322, *p* < 0.001) was the most important direct predictor of intention to dengue vaccine, followed by perceived benefit (*β* = 0.299, *t* = 6.377, *p* < 0.001) (Refer to Table 7 and Fig. 2). The findings indicated that when the respondents were inclined to have a good attitude to the dengue vaccine and viewed that it has higher benefits, they would have a positive intention to accept it. Attitude is an important factor in influencing intention whether they express likes or dislikes and support or reject anything [67]. Arham et al. [13] showed that attitude was the most important factor in expressing support for the use of Wolbachia techniques to control dengue. Besides, perceived benefit also plays a role in determining intention. Mustapa et al. [68] explained that the acceptance of new technology, especially in the field of health, disclosure of important benefits in determining intention.

**Table 7:** The relationship predicting factors that influence stakeholders’ acceptance to adopt the dengue vaccine

	*Hypothesised Path*	*Path Coefficient*	*Standard Error*	*t-values*	*p-values*	*Decision*
H1	ADV → INT	0.544	0.048	11.322	0.000***	Supported
H2	PBV → INT	0.299	0.047	6.377	0.000***	Supported
H3	PRV → INT	0.016	0.034	0.468	0.320	Not Supported
H4	PBV → ADV	0.459	0.044	10.415	0.000***	Supported
H5	PRV → ADV	0.010	0.046	0.213	0.416	Not Supported
H6	TKP → ADV	0.095	0.051	1.872	0.031*	Supported
H7	ATT → ADV	0.095	0.046	2.076	0.019*	Supported
H8	REG → ADV	0.211	0.042	4.996	0.000**	Supported
H9	TKP → PBV	0.310	0.047	6.554	0.000***	Supported
H10	ATT → PBV	0.207	0.048	4.319	0.000***	Supported
H11	REG → PBV	0.147	0.046	3.195	0.001**	Supported
H12	TKP → PRV	-0.280	0.045	6.157	0.000***	Supported
H13	ATT → PRV	-0.317	0.054	5.896	0.000***	Supported
H14	REG → PRV	-0.023	0.047	0.489	0.312	Not Supported
H15	PBV → PRV	0.082	0.057	1.427	0.077	Not Supported

**p < 0.01, *p < 0.05 (one-tailed)

Perceived benefit (*β* = 0.459, *t* = 10.415, *p* < 0.001) was the most significant direct predictor of attitude to dengue vaccine followed by religiosity (*β* = 0.211, *t* = 4.996, *p* < 0.001), attitudes to technology (*β* = 0.095, *t* = 2.076, *p* = 0.019), and trust in key players (*β* = 0.095, *t* = 1.872, *p* = 0.031) (Refer to Table 7 and Fig. 2). The results suggested that when stakeholders perceived higher benefits, clung to their religion, acknowledged that the benefits of technology outweigh risks on nature, and had a high level of trust in the key players involved in the dengue vaccine, they expressed a good attitude and accepted it. These findings indicate differences between the study of Amin and Hashim [14] and Arham et al. [13]. Arham et al. [13] also pointed out that perceived benefit and risk influenced acceptance towards Wolbachia techniques. In contrast, Amin and Hashim [14] showed that perceived benefit and trust in key players were the factors influencing stakeholders’ attitudes towards genetically modified mosquito techniques in an effort to control dengue.

Nevertheless, the stakeholders will manifest a positive attitude towards dengue control techniques when they feel the benefit. According to Amin et al. [34], the Malaysian community has firm religious beliefs, and the acceptance of the new technologies depends on their spiritual level. Conclusively, the stakeholders in this study have firm religious beliefs and do not feel that the dengue vaccine extends beyond religion. Trust in key players, such as implementers and researchers, will balance good relationships among stakeholders [69]. This notion is clearly shown in this study, where stakeholders trust key players and accept new technologies beyond the values of nature. Dengue vaccine possibly does not pose any danger to environmental health if the authorities carry out their duties properly.

Trust in key players (*β* = 0.310, *t* = 6.554, *p* < 0.001), attitudes to technology (*β* = 0.207, *t* = 4.319, *p* < 0.001), and religiosity (*β* = 0.147, *t* = 3.195, *p* = 0.001) have a positive association with perceived benefit (Refer to Table 7 and Fig. 2). This finding suggests that when stakeholders trust people who play significant roles in the dengue vaccine, are deeply attached to their religion and are more inclined to technology (negative), they benefit from the dengue vaccine. Nevertheless, attitudes to technology (*β* = -0.317, *t* = 5.896, *p* < 0.001) and trust in key players (*β* = -0.280, *t* = 6.157, *p* < 0.001) had a negative association with perceived risk (Refer to Table 7 and Fig. 2). Although they have a tendency towards technology compared to nature values, they put higher trust in key players as they feel less risk on the dengue vaccine.

The study’s findings clearly show a bipolar relationship between predictor factors with perceptions of benefit and risk, as described by Alhakimi and Slovic [70]. Mustapa et al. [71] discovered that stakeholders' acceptance of new technology is significantly influenced by high perceived benefits and low perceived risks. Therefore, the finding is further elucidated by previous studies, who showed an inverse relationship between general predictor factors such as belief in priorities, attitudes towards nature, and religion with perceptions of benefit and risk in determining the acceptance of Wolbachia and Outdoor Residual Spraying techniques [13, 18]. In conclusion, general predictor factors positively influence stakeholders’ benefits if they feel the benefits outweigh the risks. According to scholars, perceived benefit and risk are difficult to conceptualise separately because of their complex relationships that have inverse relationships [31–33].

## Conclusions

Dengue vaccination has enormous potential as a part of an integrated dengue prevention strategy to control dengue spread in Malaysia so that people can live dengue fever-free. Nonetheless, the government and authorities need to consider the collective view from the stakeholders on the dengue vaccine. This study has contributed to the stakeholders’ acceptance to adopt the dengue vaccine in Malaysia and the factors influencing their acceptance. This is the first study in Malaysia to investigate the acceptance level and the main factors the predicting intention of stakeholders to adopt the dengue vaccine in Klang Valley Malaysia. The findings are helpful to the related regulatory bodies understand the important factors influencing stakeholders’ acceptance of the dengue vaccine. The stakeholders exhibited a high level of trust in key players handling the dengue vaccine and displayed a positive attitude towards this technology. Furthermore, the stakeholders believed the vaccine did not violate religious norms and accepted the vaccine due to its benefits. Therefore, the study’s findings can serve as indicators for the decision-making process concerning implementing the dengue vaccine in Malaysia and other countries with a severe history of dengue transmission.

In addition to these valuable findings, several limitations need to be addressed in future work. First, based on the approach to data collection of a cross-sectional survey, the outcome of this study only represents a snapshot of a single timeframe. Hence, future research recommends considering a longitudinal survey for data collection. Perhaps, future research could investigate a sequential-explanatory method or qualitative approach that would include qualitative data to acquire more in-depth reasonings. In addition, the existing data was only surveyed in Malaysia. Hence it is impossible to compare consumers’ perceptions across different countries. Scholars could extend the existing model and perform a comparative analysis to examine the similarities and differences across other countries to generalize the findings (e.g., developing vs. developed countries). Moreover, future research should also account for the views of the decision-makers to shed more light on the attitude and intention of the dengue vaccine. Finally, additional factors worth investigating are knowledge, perceived susceptibility, and severity and how such factors could potentially influence acceptance of the dengue vaccine as past studies have reported the influence of these factors in predicting vaccine uptake intentions. In conclusion, the dengue vaccine is a good effort, but continuous research must be conducted to ensure universal safety.

## Supplementary Information

**Additional file 1: Supplementary Material 1.** Measurement Items.

## Data Availability

All relevant data are within the manuscript and the measurement items was included in supplementary file.

## Abbreviations

ADV: Attitude to dengue vaccine
ATT: Attitudes to technology
AVE: Average variance extracted
CR: Composite reliability
CYD-TDV: Tetravalent, live attenuated, chimeric dengue vaccine (Dengvaxia)
DENV: Dengue virus
HTMT: Heterotrait-monotrait
INT: Intention to dengue vaccine
NFI: Normed fit index
PBV: Perceived Benefit
PLS: Partial least squares
PRV: Perceived Risk
REG: Religiosity
SEM: Structural equation modelling
SPSS: Statistical package for the social sciences
SRMR: Standardised root mean square residual
TKP: Trust in key players
VIF: Variance inflation factor
