# Supplementary material for: “To do, or not to do?”: determinants of stakeholders’ acceptance on dengue vaccine using PLS-SEM analysis in Malaysia
Source: BMC Public Health. 2022 Aug 19;22:1574. doi: 10.1186/s12889-022-13967-3 (PMC9388355; doi:10.1186/s12889-022-13967-3)
Supplement: Supplementary file 1 — Additional file 1: Supplementary Material 1. Measurement Items. [file 12889_2022_13967_MOESM1_ESM.docx]

**Supplementary Material 1:** Measurement Items

| **Factors/Items** |
| --- |
| **Intention to Dengue Vaccine**  INT1: I am willing to support dengue vaccine if it can combat dengue. |
| INT2: I am willing to support dengue vaccine if it is beneficial to my health and the health of other people. |
| INT3: I am willing to support dengue vaccine if there are no other better alternatives. |
| INT4: I am willing to support dengue vaccine if there are no other better alternatives. |
| INT5: I am willing to support dengue vaccine if they have been proven effective to combat dengue in other areas. |
| INT6: I am willing to support dengue vaccine if the government can ensure the effectiveness of it. |
| **Attitude to Dengue Vaccine**  ADV1: Dengue vaccine should be scaled up. |
| ADV2: Government should provide more financial support to researchers and industries in developing the dengue vaccine. |
| ADV3: Dengue vaccine help government to decrease community’s fatality. (casualities in the community). |
| ADV4: Dengue vaccine is necessary. |
| ADV5: Dengue vaccine is encouraged. |
| **Perceived Benefit**  PBV1: Dengue vaccine will enhance the quality of life. |
| PBV2: Dengue vaccine is useful to the Malaysian society. |
| PBV3: Dengue vaccine is useful in preventing dengue fever. |
| PBV4: Dengue vaccine is effective to eradicate dengue. |
| PBV5: Dengue vaccine is beneficial to me and my family’s health. |
| PBV6: The benefits of the dengue vaccine to people outweigh their risks. |
| PBV7: Whatever the risks of the dengue vaccine will be dealt with in future research |
| **Perceived Risk**  PRV1: Level of worries about the unknown effects of the dengue vaccine? |
| PRV2: Any harmful effects from using the dengue vaccine will only manifest itself after long term duration? |
| PRV3: Dengue vaccine pose threat to future generation. |
| PRV4: Dengue vaccine may give rise to unknown consequences. |
| PRV5: Any danger from the dengue vaccine may cause a major catastrophe to Malaysian society. |
| PRV6: How worried are you about the potential risks of the dengue vaccine to your health and you family’s health? |
| PRV7: Adverse effects from the dengue vaccine are harmful. |
| **Trust in Key Actors**  TKP1: Scientists have done a good job for society |
| TKP2: Industries have done a good job for society. |
| TKP3: Government have done a good job for society. |
| **Attitudes to Technology**  ATT1: Technology has made humans have less respect for nature. |
| ATT2: Technology has made humans more focused on profits. |
| ATT3: Technology has destroyed the value of humanity. |
| ATT4: Technology has made human beings limitless to the point of losing humanity. |
| ATT5: Technology has made humans threaten the balance of nature. |
| ATT6: Technology has made human beings a problem for urban life. |
| **Religiosity**  REG1: Religion is important in my life. |
| REG2: Religious views are important when I have to make decisions about controversial issues. |
| REG3: Praying is important in my life. |
| REG4: Reading scriptures is important in my life. |
| REG5: Religion is especially important to me because it answers many questions about the meaning of life. |
| REG6: What religion offers me most is comfort when sorrows and misfortune strike. |
| REG7: I try hard to live all my life according to my religious beliefs. |
| REG8: Nothing can occur without God’s involvement in the process. |
